# Targeting PDK1 with dichloroacetophenone to inhibit acute myeloid leukemia (AML) cell growth

**DOI:** 10.18632/oncotarget.6366

**Published:** 2015-11-22

**Authors:** Lijun Qin, Yun Tian, Zhenlong Yu, Dingbo Shi, Jingshu Wang, Changlin Zhang, Ruoyu Peng, Xuezhen Chen, Congcong Liu, Yiming Chen, Wenlin Huang, Wuguo Deng

**Affiliations:** ^1^ Department of Pediatrics, Sun Yat-sen Memorial Hospital, Sun Yat-sen University, Guangzhou, China; ^2^ Sun Yat-sen University Cancer Center, Guangzhou, China; ^3^ State Key Laboratory of Oncology in South China, Guangzhou, China; ^4^ Collaborative Innovation Center of Cancer Medicine, Guangzhou, China; ^5^ Guangzhou Women and Children's Medical Center, Guangzhou, China; ^6^ Institute of Cancer Stem Cell, Dalian Medical University, Dalian, China; ^7^ Guangdong Provincial No. 2 People's Hospital, Guangzhou, China; ^8^ State Key Laboratory of Targeted Drug for Tumors of Guangdong Province, Guangzhou Double Bioproduct Inc., Guangzhou, China

**Keywords:** PDK1, AML, apoptosis, autophagy, BCL-xl

## Abstract

Pyruvate dehydrogenase kinase-1 (PDK1), a key metabolic enzyme involved in aerobic glycolysis, is highly expressed in many solid tumors. Small molecule compound DAP (2,2-dichloroacetophenone) is a potent inhibitor of PDK1. Whether targeting PDK1 with DAP can inhibit acute myeloid leukemia (AML) and how it works remains unknown. In this study, we evaluated the effect of inhibition of PDK1 with DAP on cell growth, apoptosis and survival in AML cells and identified the underlying mechanisms. We found that treatment with DAP significantly inhibited cell proliferation, increased apoptosis induction and suppressed autophagy in AML cells *in vitro*, and inhibited tumor growth in an AML mouse model *in vivo.* We also showed that inhibition of PDK1 with DAP increased the cleavage of pro-apoptotic proteins (PARP and Caspase 3) and decreased the expression of the anti-apoptotic proteins (BCL-xL and BCL-2) and autophagy regulators (ULK1, Beclin-1 and Atg). In addition, we found that DAP inhibited the PI3K/Akt signaling pathway. Furthermore, we demonstrated that PDK1 interacted with ULK1, BCL-xL and E3 ligase CBL-b in AML cells, and DPA treatment could inhibit the interactions. Collectively, our results indicated that targeting PDK1 with DAP inhibited AML cell growth via multiple signaling pathways and suggest that targeting PDK1 may be a promising therapeutic strategy for AMLs.

## INTRODUCTION

Acute myeloid leukemia (AML) is characterized by the deregulated proliferation of immature myeloid cells.[1] Nearly 80% of adult leukemias are AMLs with a median age at diagnosis of 65 years.[1] The majority of AML patients who receive intensive chemotherapy can achieve complete remission. However, the relapse rates are high, and the overall five-year survival rate is 20%. Patients who are older than 60 years have even poorer prognoses (5-year survival rates <10%).[1] In recent years, inhibitors of FMS-like tyrosine kinase 3 (FLT3) have emerged as promising treatment options for AML. However, only 35% of AML patients have acquired a FLT3 mutation.[2]Therefore, it is of importance to discover and identify new key regulators that are widely expressed in the majority of AML patients.

Nearly 80 years ago, Otto Warburg discovered that the majority of tumor cells favor aerobic glycolysis even when oxygen is plentiful. This phenomenon was later termed the Warburg effect. Warburg hypothesized that this metabolic dysfunction caused the development of tumors.[3] The most sensitive clinical diagnostic tool for cancer is positron emission tomography (PET) because aerobic glycolysis is crucial for most tumor cells. Recently, the study of tumor metabolism has been revived. Pyruvate kinase M2 (PKM2) is expressed predominantly in tumor cells [4, 5] and is important for tumor growth because it is the rate-limiting enzyme in the glycolysis pathway. PKM2 catalyzes the conversion of phosphoenolpyruvate and ADP into pyruvate and ATP. Other researchers discovered that the inhibition of key kinases in the glycolysis pathway has strong anti-tumor effects, [6, 7] and the inhibition of PKM2 markedly induces apoptosis in breast tumor cells.[8]

Pyruvate dehydrogenase kinase-1 (PDK1) is the key metabolic enzyme involved in aerobic glycolysis, and it has been found to be overexpressed in many tumors. [9, 10] PDK1 acts as a gate keeper that regulates the flux of pyruvate from the cytoplasm into the mitochondria. [9] By inhibiting the pyruvate dehydrogenase complex, PDK1 decouples glycolysis from oxidative phosphorylation. PDK1 overexpression may inhibit mitochondrial function, which may lead to resistance to apoptosis. The inhibition of PDK sensitizes both wild type and BCL-2-overexpressing cancer cells to radiation by interacting with BCL-2. [11] Recent study has also demonstrated that the prognosis is poor for gastric cancer patients who have high levels of PDK1.[12]

PDK inhibitor dichloroacetate (DCA) has been reported to inhibit PDK1 signaling and exert strong anti-tumor effects in glioblastoma. Therefore, we inferred that PDK1 is important for tumor cell survival. Based on the results of previous studies [11, 13, 14]. DCA is a relatively weak inhibitor of PDK1, and millimolar (mM) concentrations of DCA are required to achieve the desired effects. Some studies has shown DCA is toxic (ie neuropathy), which has resulted in some clinical trials being halted [15, 16]*.* Because it is difficult to avoid off-target effects at mM concentrations, it is necessary to identify stronger inhibitors. Importantly, 2,2-dichloroacetophenone (DAP) is a much more potent inhibitor of PDK1. It is effective at concentrations in the micromolar (μM) range.

In established cancer cells, autophagy is often induced as an alternative source of energy and metabolites. [17] When cancers are treated with HDAC inhibitors or rapamycin, autophagy is often induced as a pro-survival strategy.[18, 19] These previous studies suggested that inhibiting autophagy could sensitize cancer cells to HDAC inhibitors or rapamycin. Moreover, Chen *et al.*[19] discovered that the suppression of ATG5, ATG6, or ATG7 and co-treatment with bafilomycin A1 sensitized various tumors to several anti-cancer drugs and therapies.

The PI3K/Akt/mTOR pathway is one of the major cellular survival pathways. The constitutive activation of the PI3K/Akt/mTOR pathway has been reported to be a common feature in AML patients. [20, 21] This pathway also controls the membrane ATP-binding cassette (ABC) transporter, multidrug resistance associated protein 1. For these reasons, many small molecules, such as rapamycin and PI-103, [22, 23] have been developed to target this pathway. However, for the most part, the results of the clinical trials that have been performed with these compounds have been disappointing. [24, 25] The outcomes of these clinical trials suggest that tumor cells may bypass the PI3K/Akt pathway. Thus, there is an urgent need to identify a more promising target that is essential for cancer cell survival. Akt also regulates aerobic glycolysis. [26] 3-Phosphoinositide-dependent protein kinase-1 may phosphorylate and activate Akt, [27] but it is unclear if pyruvate dehydrogenase has an effect on the PI3K/Akt pathway.

In this study, we investigated the role of targeting PDK1 with DAP in the regulation of the growth and survival of AML cells and identified the underlying mechanisms of actions. Our study suggests that PDK1 may be a promising therapeutic target for AML.

## RESULTS

### DAP suppressed cell proliferation *in vitro* in AML cell lines

**Figure 1:**
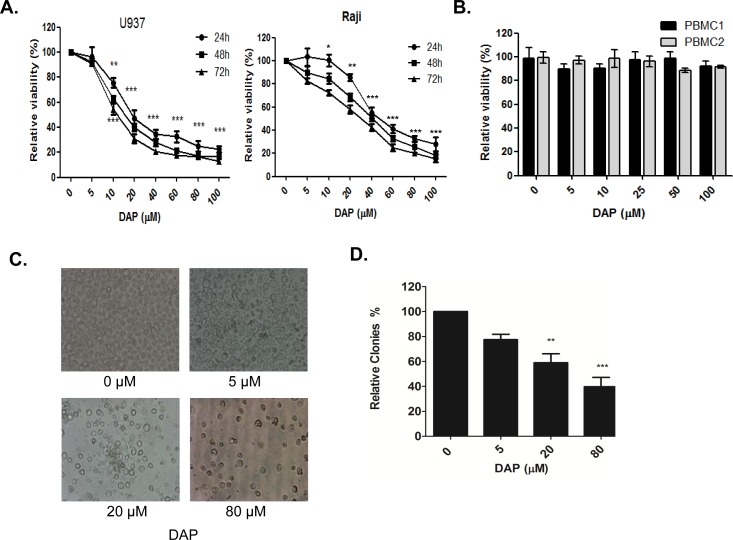
DAP inhibited AML cell growth **A**. The U937 and Raji cells were treated with the increasing concentrations of DAP (0, 5, 10, 20, 40, 60, 80 and 100 μM) for 24, 48 or 72 h. Cell viability was measured using the CCK-8 assay. **B**. The normal blood cells (PBMCs) from healthy donors, were treated with the increasing concentrations of DAP (0, 5, 10, 20, 40, 60, 80 and 100 uM) for 24 h. Cell viability was measured using the CCK-8 assay. **C**. AML U937 cells were treated with the increasing concentrations of DAP for 24 h. Microscopy analysis was used for analyze the number of cells. **D**. Counts of clones in the soft agarose gel under a microscope (10x magnification) after 4 weeks scoring 5 different fields for each DAP concentration. All assays were repeated three times, and statistical significance was tested by SPSS11.0 (* represents *P*<0.05, ** represents *P*<0.001, *** represents *P*<0.0001, significant differences between the DAP-treatment groups and the non-treatment control groups, which was set as 100% viability at 24, 48 and 72 hours, respectively.

We first evaluated the effects of PDK1 inhibitor DAP on cell growth in AML U937 and Raji cell lines. The cells were treated with the increasing concentrations of DAP at 0, 5, 10, 20, 40, 60, 80 and 100 μM for 24, 48 or 72 hours. The cell viability was measured using a CCK-8 assay. As shown in Figure 1A, DAP at 5 μM slightly inhibited cell growth, but DAP at 10 μM or higher concentrations significantly inhibited cell viability in a dose-dependent manner. The IC50 values were 14.0 μM for U937 cells and 24.4 μM for Raji cells. However, DAP treatment had no significant inhibition on cell viability in the normal blood cells (PBMCs) (Figure 1B). Because the U937 cell line was more sensitive to DAP than Raji cell line, we chose this AML cell line as a model to study the molecular mechanism by which DAP targeted PDK1 to inhibit AML growth. Microscopy analysis also revealed that the number of cells decreased in a concentration-dependent manner (Figure 1C). We also examined the effects of PDK1 inhibition on colony formation using soft agar colony formation assays. The number of colonies decreased as the concentration of DAP increased (Figure 1D).

### DAP suppressed tumor growth *in vivo* in an AML mice model

**Figure 2:**
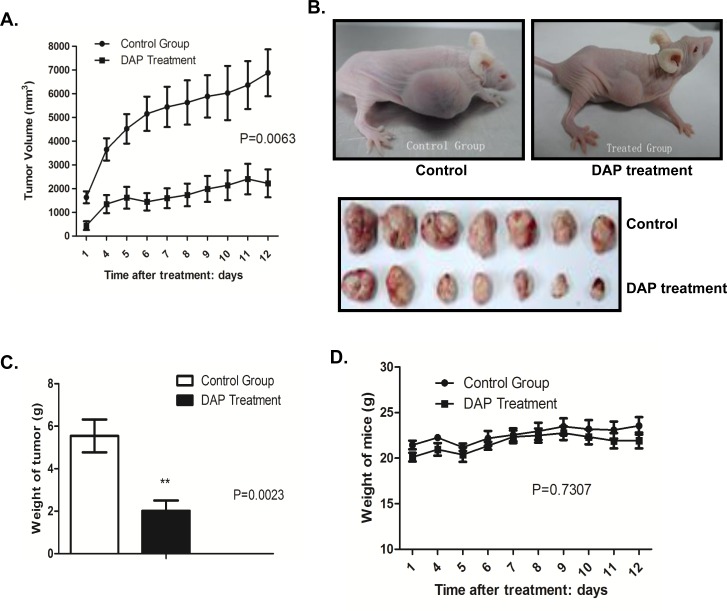
DAP suppressed tumor growth in an AML mouse model U937 cells were injected subcutaneously into the nude mice, and the visible tumors developed at the injection sites after 4 days. DAP was then subcutaneously injected for two weeks. **A**. Tumor growth curve. The record of tumor growth started from the time point when the tumors formed and were treated with DAP. **B**. Mice and tumors. **C**. Tumor weights. **D**. Body weight weights of the mice.

To confirm the inhibition of DAP in AML cell growth and survival, we analyzed the effects of DAP treatment on tumorigenicity *in vivo* using a AML xenograft mouse model. U937 cells were injected subcutaneously into the nude mice, and the visible tumors developed at the injection sites after 4 days. DAP was then subcutaneously injected for two weeks. As shown in the growth curve in Figure 1A, DAP treatment markedly suppressed tumor growth (Figure 2A). At 12 days, the tumors were taken out and weighted. DAP effectively inhibited the tumor volumes (Figure 2B) and tumor weights (Figure 2C) as compared to the control group (*p*=0.0023). However, treatment with DAP did not affect significantly the body weight weights of the mice (*p*=0.7307) (Figure 2D).

### DAP induced apoptosis and inhibited PDK1 experssion in AML cells

**Figure 3:**
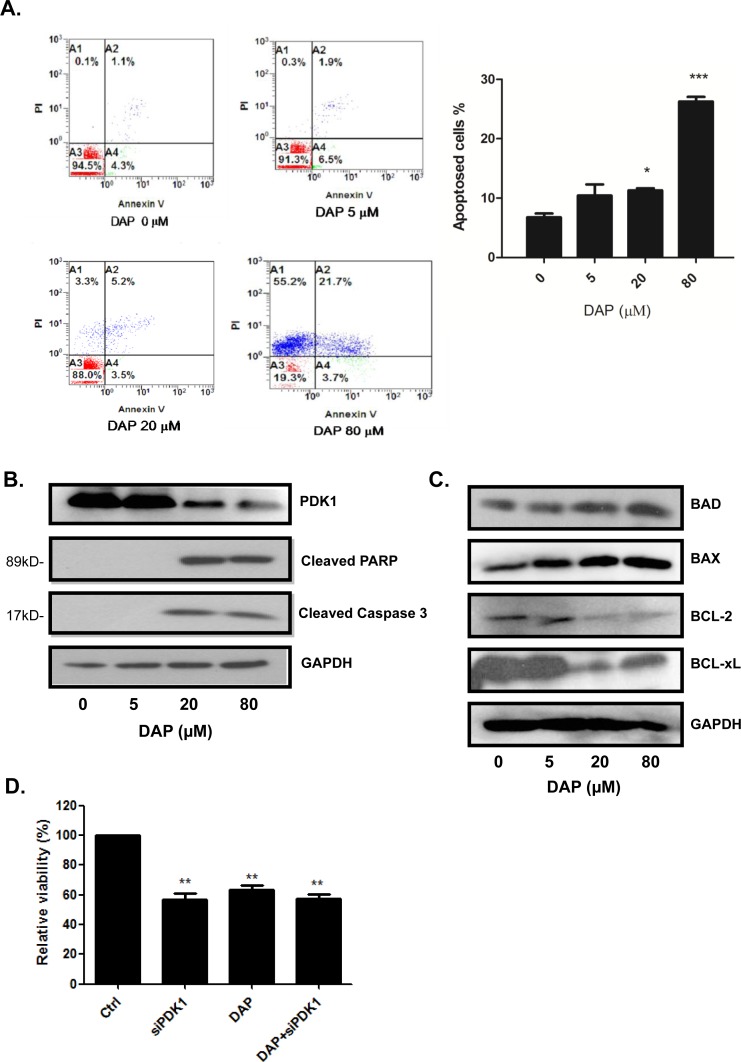
DAP induced apoptosis and inhibited PDK1 signaling in AML cells U937 cells were treated with DAP for 24 h, and apoptosis was detected by Annexin V-PI staining **A**. The expression of cleaved PARP, caspase 3 **B**., and the levels of BCL-2, BCL-xL (anti-apoptotic proteins), BAX (pro-apoptotic protein) and BAD (BH3-only protein) **C**. were detected by Western blot. **D**. U937 cells were pre-treated with PDK1 siRNA for 48 hours and followed treatment with or without DAP for 24 h. Cell viability was measured using the CCK-8 assay. The data are presented as the mean ± S.D. of three separate experiments. (*, *P*<0.05; **, *P*<0.001; ***, *P*<0.0001).

We next analyzed the effect of DAP on apoptosis by flow cytometry. Based on the results of our preliminary screening using the CCK-8 assay, we selected 5, 20, and 80 μM DAP as the representative doses for the next experiments. After 24 h of exposure to DAP, U937 cells were subjected to Annexin V-PI double staining and analyzed by flow cytometry. DAP treatment resulted in a significant increase in the proportion of Annexin V-positive apoptic cells (Figure 3A). Interestingly, we found that DAP induced late apoptosis in a dose-dependent manner but not any change for early apoptosis (Figure 3A). In addition, DAP induced the cleavage of the pro-apoptotic proteins PARP and caspase-3 in U937 cells (Figure 3B). We also found that DAP treatment decreased the protein levels of the anti-apoptotic BCL-2 family proteins, namely BCL-XL and BCL-2, and up-regulated the expression of the apoptotic proteins BAX (Figure 3C). Moreover, we showed that in U937 cells treatment with DAP markedly inhibited the expression of PDK1 protein (Figure 3B), implying that DAP inhibited AML cell growth and induced apoptosis through PDK1 signaling pathway.

To further confirm whether the influence of DAP on cells survival was through targeting PDK1, the U937 cells were pre-transfected with PDK1 siRNA for 48 h to inhibit the PDK1 singaling first, and then followed by the addition DAP. After 24 h, cell proliferation was analyzed by CCK-8 assay. As shown in Figure 3D, knockdown of PDK1 by siRNA or treatment with DAP alone considerably inhibited cell proliferation. However, the addition of DAP did not dramatically increased the proliferation inhibition induced by PDK1 siRNA in AML cells as compared with the treatment with DAP alone, implying that DAP regulated AML cell growth by targeting PDK1 signaling.

### DAP suppressed autophagy in AML cells

**Figure 4:**
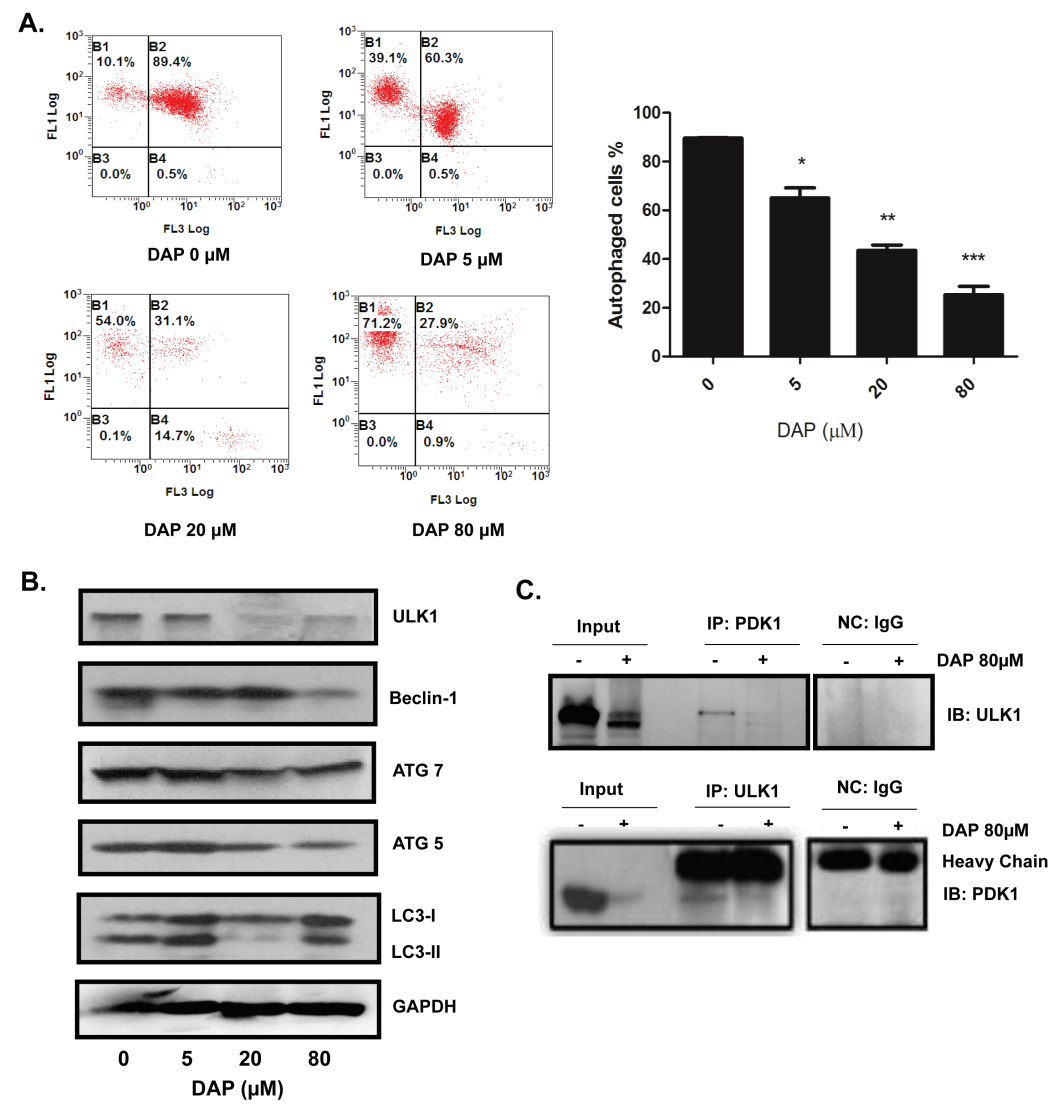
DAP inhibited autophagy in AML cells U937 cells were treated with DAP for 24 h. **A**. The total level of autophagy was detected using acridine orange staining. **B**. The autophagy regulator proteins ULK1, Beclin-1, LC3П, Atg5 and Atg7 were detected by Western blot. **C**. The interaction of PDK1 with ULK1 was detected by IP and Western blot assays.

Cell autophagy plays an important role in tumor growth. We next tested whether the DAP-mediated inhibition of AML cell growth is due to the regulation of autophagy. The U937 cells were treated with increasing concentrations of DAP for 24 h and autophagy was detected using acridine orange staining and flow cytometry. The results showed that the number of autophagic vesicles decreased as the concentration of DAP increased (Figure 4A). The changes in the expression levels of autophagy-regulating proteins were measured by Western blotting analyses. The expression of the early stage autophagy regulators, namely ULK1, Beclin-1, Atg 5 and Atg 7, was significantly inhibited by DAP in a dose-dependent manner, and also the LC3-I converted to II (Figure 4B). We then performed Co-IP to test if PDK1 could target and interact with the autophagy regulatory protein ULK1 and DAP treatment could inhibited this interaction. As shown in Figure 4C, a clear interaction between PDK1 and ULK1 was detected in AML cells, and treatment with DAP considerably inhibited the expression of ULK1, thereby decreasing the amount of ULK1 protein which interacted with PDK1.

### DAP inactivated PI3K/Akt signaling pathway in AML cells

**Figure 5:**
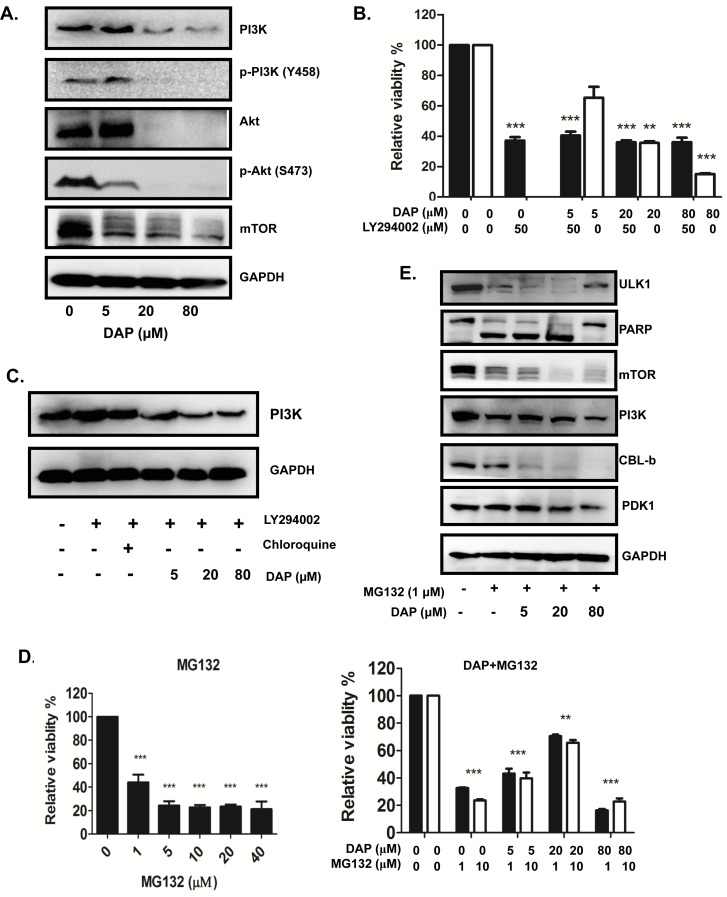
DAP inhibited PI3K/Akt signaling pathway in AML cells **A**. U937 cells were treated with DAP for 24 h, and the protein levels of Akt, PI3K, mTOR and Erk and their phosphorylation levels were detected by Western blot. **B**. U937 cells were treated with 50 μM LY294002 (an inhibitor of PI3K and mTOR) in combination with DAP for 24 h. The cell viability was analyzed. **C**. U937 cells were treated with DAP in combination with LY294002 or chloroquine, the protein level of PI3K was detected with Western blot.

The PI3K/Akt signaling pathway is implicated in AML cell growth and survival. We next determined the effect of DAP on the regulation of the key molecules involved in the PI3K/Akt pathway in the Akt-overexpressing U937 cells. The cells were treated with DAP at the doses of 5, 20, or 80 μM for 24 h and the proteins were detected by Western blot assay. As shown in Figure 5A, the levels of Akt, phospho-Akt, PI3K, phospho-PI3K and mTOR proteins were markedly inhibited by DAP. To verify the PI3K/Akt signaling pathway is involved in the DAP-mediated inhibition of AML cells. We pre-treated U937 cells with the PI3K inhibitor LY294002 at 50 uM to block the PI3K/Akt signaling pathway and followed the treatment with DAP. As shown in Figure 5B, treatment with LY294002 alone significantly inhibited U937 cell proliferation, so we chosen the dose of 50 uM for our next experiments. The addition of DAP did not dramatically affect the LY294002-mediated inhibition of proliferation compared to the treatment with LY294002 or DAP alone. Moreover, neither LY294002 nor chloroquine, an inhibitor of autophagy, decreased the level of PI3K protein expression. Only DAP treatment decreased PI3K protein expression levels (Figure 5C). Based on these observations, we postulated that PDK1 stabilized PI3K and that the inhibition of PDK1 promoted PI3K degradation. To text this possibility, we pre-treated U937 cells with the proteasome inhibitor MG132 and followed the treatment with DAP. As shown in Figure 5D, MG132 could inhibit U937 cell proliferation, however, combination with DAP, there was no obvious difference between 1 μM MG132 and 10 μM MG132. And also, we detected the protein levels of PI3K and PDK1, as well as relative proteins. As shown in Figure 5E, pre-treatment with MG132, the degradation of PI3K and PDK1 was significantly decreased.

### DAP inhibited the interaction of PDK1 with CBL-b, Akt and BCL-xL

**Figure 6:**
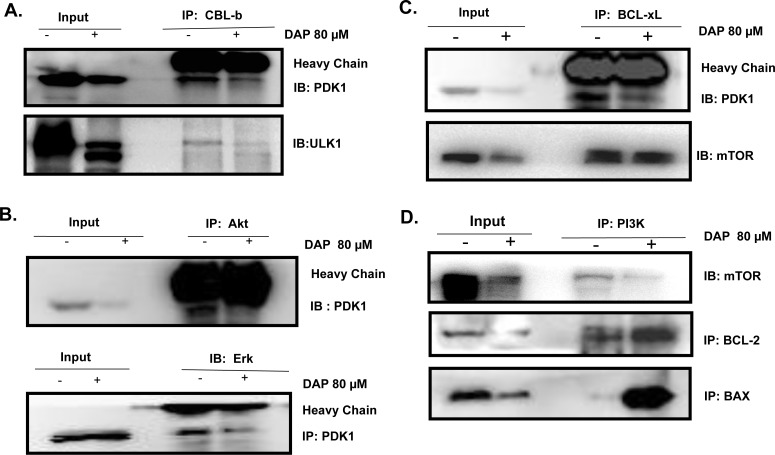
DAP inhibited the interaction of PDK with CBLb, Akt and BCL-xL in AML cells U937 cells were treated with DAP for 24 h, and **A**. the interaction of CBL1 with PDK1 or ULK1; **B**. the interaction of Akt or Erk with PDK1; and C. the interaction of BCL-xL with PDK1 or mTOR; and the interaction of PI3K with BCL-2 or BAX, were detected by IP and Western blot.

We next determined the potential molecular targets of PDK1 which interact with PDK1 and assessed the effect of DAP on their interactions in AML cells by Co-IP assay. We found that the E3 ligase CBL-b could interact with PDK1 and the autophagy regulatory protein ULK1 (Figure 6A). Treatment with DAP effectively abolished the interaction of CBL-b with PDK1 or ULK1 (Figure 6A). We also detected the interaction of PDK1 with Akt or Erk and found that treatment with DAP considerably inhibited the expression of Akt, thereby decreasing the amount of Akt protein which interacted with PDK1 (Figure 6B).

BCL-xL is involved in AML proliferation. We next detected the interaction between PDK1 and BCL-xL in AML cells. After treatment with DAP, the interaction between PDK1 and BCL-xL was decreased, but the interaction between BCL-xL and mTOR did not change (Figure 6C). Both BCL-2 (anti-apoptotic member of the BCL-2 family) and BAX (pro-apoptotic BCL-2 family member) interacted with PI3K. After exposure to DAP, the interactions between PI3K and both BCL-2 and BAX increased dramatically (Figure 6D).

## DISCUSSION

In the present study, we demonstrated that the DAP strongly inhibited cell growth via inhibiting PDK1 in many different AML cell lines, which suggest that PDK1 inhibition has a broad spectrum of anti-tumor effects. We found that the canonical PI3K/Akt survival pathway was also inhibited by DAP, and this inhibition decreased the post-translational modifications and reduced the expression levels of key regulatory proteins. In our study, we also demonstrated that the CBL-b E3 ligase interacted with PDK1 and ULK1 and that CBL-b levels were not significantly altered when the cells were exposed to DAP.

Autophagy protects tumor cells from death when they are challenged with starvation or other distresses [29, 30]. ULK1 (Unc-51 like kinase1) is a key molecule involved in triggering autophagy initiation. ULK1 is suggested to have a significant contribution in controlling autophagy. [31] In our study, we showed that DAP induced cell apoptosis and suppressed cell autophagy though inhibiting PDK1 and ULK1 expression, respectively. Recently, it has also been shown that both PDK1 and ULK1 affect the function of mitochondria. [9, 32] mTOR is known to suppress ULK1, and BCL-2 is known to suppress Beclin-1. [33, 34] Therefore, autophagy may be activated or enhanced when PI3K/mTOR and BCL-2 are strongly inhibited. Here, we hypothesized that PDK1 might interact with these factors, and then have effects on mitochondria. However, our results indicated that DAP decreased autophagy. The protein levels of ULK1 and Beclin-1 were reduced when PDK1 was inhibited, indicating that the PDK1-mediated regulation of autophagy may be independent of PI3K/mTOR and BCL-2. The results of the Co-IP experiments verified our hypotheses and showed that PDK1 interacted with ULK1. Because PDK1 regulates the pyruvate dehydrogenase complex, PDK1 may also regulate autophagy. Both of these pathways provide important building blocks for tumor cells, and these two pathways require coordination. Therefore, PDK1 may be one of these signal hubs.

When LY294002 was added to the cells, the DAP-mediated inhibition of proliferation was blocked, thereby indicating that PDK1 required PI3K and mTOR activity to carry out its function. Furthermore, neither LY294002 nor chloroquine decreased the protein levels of PI3K, but DAP reduced the levels of PI3K. Based on these observations, we postulated that PDK1 stabilized PI3K and that the inhibition of PDK1 promoted PI3K degradation. However, we discovered that the inhibition of PDK1 reduced the protein levels of PI3K, Akt, mTOR and Erk. We found it is difficult to explain using a model whereby PDK1 stabilizes PI3K. Therefore, we postulated that PDK1 decreased the degradation of some onco-proteins, such as Akt. When the MG132 proteasome inhibitor was added, the degradation of PI3K and PDK1 was significantly decreased, thereby suggesting that PDK1 may be coupled with the ubiquitin degradation system and act as a brake. In addition, we found that both PDK1 and ULK1 interacted with the CBL-b E3 ligase. Based on our data, we suggest that PDK1 blocks the E3 ligase-dependent degradation of key oncoproteins.

Elstrom *et al*. [26] reported that Akt-expressing cancer cells up-regulate the rate of aerobic glycolysis, and we demonstrated that Akt directly interacted with PDK1, which may explain why tumors shut down their mitochondria to achieve apoptotic resistance and up-regulate survival proteins, such as Akt. PDK1 may be located at a pivotal position in coordinating these two tasks.

Following the induction of apoptosis, the interactions between PDK1 and Erk, Akt, and BCL-xL decreased, but BCL-xL remained bound to mTOR. The DAP-induced decreased expression of BCL-xL may have disrupted the interaction between PI3K and mTOR. Our co-IP data suggested that PI3K may have caused the release of BCL-2 from the mitochondria, assisted in Bax oligomerization, and released procaspase-8.

**Figure 7:**
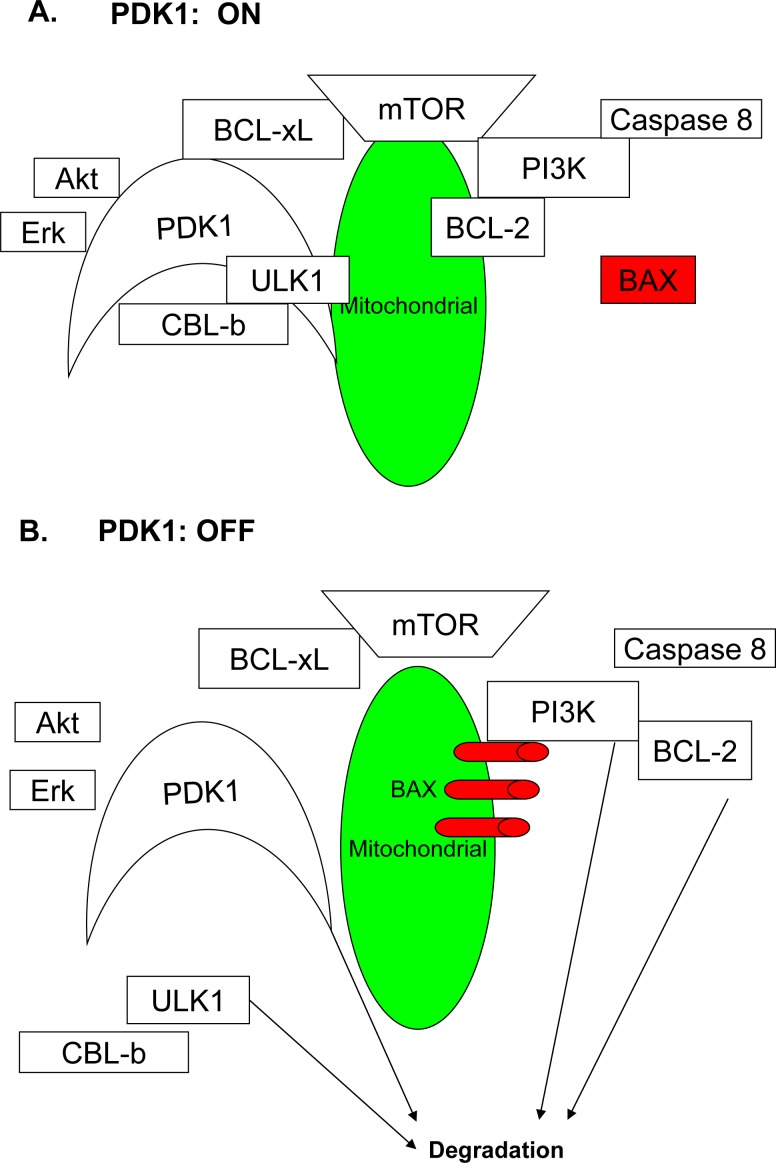
The model for the role of PDK1 in AML cell growth **A**. When PDK1 is active, it blocks the CBL-b E3 ligase and prevents the degradation of oncoproteins, such as ULK1, Akt, PI3K, and BCL-xL, by the proteasome. PI3K binds to BCL-2 and blocks BAX oligomerization. **B**. When PDK1 is inhibited by DAP, the CBL-b E3 ligase releases from PDK1, and the ULK1, Akt, PI3K, and BCL-xL oncoproteins are degraded. PDK1 itself is also degraded. Meanwhile, PI3K releases BCL-2, translocates BAX to the mitochondria and allows the BAX oligomerization, thereby inducing apoptosis.

PDK1 not only acts as a metabolic enzyme but also as an oncogene protecting tumor cells from apoptosis. Based on the results of our study, we inferred that PDK1 protected BCL-2, BCL-xL, and PI3K/Akt/mTOR from degradation and that the persistent stabilization of these proteins made the tumor cells resistant to apoptosis, thus conferring survival advantages. PI3K was one of the proteins protected from degradation, and PI3K was found to be a major mediator of PDK1 activation (see Figure 7).

Inhibition of the pyruvate dehydrogenase complex by PDK1 is crucial to maintain energy homeostasis. Dichloroacetate (DCA) is a relatively weak inhibitor of PDK1 and has been shown to inhibit tumor growth in glioblastoma. However, some studies with DCA proved this compound to be toxic (ie neuropathy). In our study, we demonstrated that 2,2-dichloroacetophenone (DAP) is a much more potent inhibitor of PDK1. It is effective at concentrations in the micromolar (μM) range. We did not anticipate that the inhibition of PDK1 would cure cancer. Instead, we were searching for potential processes and targets that are vital to cancer based on the literature. We believed that PDK1 may be one such target and that tumor metabolism was one of these vital processes. PDK1 inhibition has been shown to have powerful and broad spectrum anti-tumor effects *in vitro*, and these effects are relatively unique compared to tyrosinase inhibitors. Our animal experiments showed that DAP inhibited tumor growth as compared to controls, but was unable to completely inhibit tumor growth. Additional research is required, and more connections between apoptosis and tumor metabolism must be identified before PDK1 inhibition can be used in a clinical setting.

Based on the data obtained in the present study, we propose the model depicted in Figure 7. In tumor cells, PDK1 acts as a brake blocking the CBL-b E3 ligase, thereby preventing the degradation of key oncoproteins, such as PI3K and BCL-xL, by proteasome. When PDK1 is inhibited by DAP, the inhibition of the CBL-b E3 ligase is decreased, which promotes the degradation of PDK1. Following PDK1 degradation, the levels of Akt, PI3K, BCL-xL, BCL-2, mTOR, and ULK1 are also decreased, and the interactions between these proteins become increasingly weaker. In addition, BAX loses its abilities to inhibit BCL-2 and BCL-xL, translocate to the mitochondria, and induce apoptosis. Because ULK1, the key regulator of autophagy, is reduced, it becomes difficult for tumor cells to up-regulate autophagy and restore survival.

Previous research has been found that PDK1 is associated with various types of cancers including melanoma, breast cancer, lung cancer, stomach cancer, prostate cancer, ovarian cancer, and pancreatic cancer [35]. Most researchers adopted tumortransplantation mouse model to study PDK1 expression on tumor growth. In this study, we also analyzed the effect of DAP treatment on tumorigenicity *in vivo* using a U937 cells AML xenograft mouse model. Our data showed that DAP treatment markedly suppressed tumor growth. However, the deviation of tumors in the treatment group are much smaller than those in the control group at the end point (Day 12), while the deviation of tumors in the control group is smaller than the DAP treatment group. That is because the tumor size for each mouse in the DAP treatment group was small and we measured the tumor size outside the skin of mice, the deviation in the DAP treatment group was smaller than those in the control group.

In addition, in our study, at day-1 after treatment, the tumor volumes of xenograft are a little bit different between the control group and treatment group. The tumor volumes of the control group have been over 1000 mm3 after 4-day cell injection and drug injection for 2 weeks, and rapidly increased to 6000 mm3 within 12 days. Therefore, it would be better if the record of tumor growth shown in the graph started from the time point when the tumors formed and were treated with DAP. Moreover, an *in vivo* model such as NRas/Bcl-2 AML may be more relevant than U937 xenografts and the Western blot data from tumors for the same signaling pathways would strengthen this paper. Oxidative stress in AML and drug response is of key interest in the field. The assays to address the oxidative stress response and/or DNA damage to DAP would be important to investigate the metabolic effects of PDHK inhibition on these cells. Thus, the further study is needed in the future.

We conclude that targeting PDK1 by DAP inhibited cell proliferation and induced apoptosis induction in AML cells*.* We also showed that DAP suppressed the expression of the anti-apoptotic proteins and autophagy regulators and PI3K/Akt signaling pathway. Moreover, we demonstrated that PDK1 interacted with ULK1, BCL-xL and E3 ligase CBL-b in AML cells, and DPA treatment could inhibit the interactions (see Figure 7).

## MATERIALS AND METHODS

### Cell culture and chemicals

BCL-2- and Akt-overexpressing human myeloid leukemia U937 cell lines were maintained in RPMI 1640 medium supplemented with 10% fetal calf serum (FCS, Hyclone, Logan, UT). 2, 2-dichloroacetophenone (DAP) was purchased from Sigma (St. Louis, MO). Stock solutions of the drug (1 mM and 1 M) were prepared in dimethyl sulfoxide (DMSO), stored as small aliquots at −20°C and diluted as needed in cell culture medium.

### Cell counting Kit-8 assay

The Cell Counting Kit-8 assay was used to determine cell proliferation and cytotoxicity. Briefly, 10000 cells/well were plated in 96-well plates and treated with various concentrations of DAP. The cells were then grown for an additional 24, 48, and 72 h, and the Cell Counting Kit-8 assay was used according to the manufacturer's recommended instructions (Dojindo, Japan). The effect on cell viability was assessed as the percent cell viability compared to the un-treated control group, which were arbitrarily assigned 100% viability at 24, 48 and 72 hours, respectively. IC50 was calculated by Graphpad Prism 5.0.

### Soft agarose cloning assay

Low-melting agarose was dissolved in pure water at 1.2 and 0.7%, sterilized using an autoclave, and then warmed at 42°C in a water bath. One ml of 2x RPMI 1640 was transferred to each well of a 6-well plate, and 1 ml of 1.2% agarose was then added. After these two solutions were mixed, 500 μl of 0.7% agarose and 500 μl of RPMI 1640 containing 500 cells were pipetted into each well. Two weeks later, the number of colonies was counted.

### Analysis of apoptosis

The DAP-treated cells (1× 10^5^ cells/ml) were stained with Annexin V-FITC using an Annexin V/Dead Cell Apoptosis Kit (Invitrogen, USA). The cells were washed twice in cold PBS and resuspended in binding buffer. This suspension (100 μl) was stained with 5 μl of Annexin V-FITC, gently vortexed, and incubated in the dark for 15 min at room temperature. Finally, 10 μl of PI was added, and the mixture was incubated in the dark for five min at room temperature. After the addition of 400 μl of binding buffer to each tube, the cells were analyzed using Cytomics FC500 Flow Cytometer (Beckman Coulter).

### Detection of autophagy

The cells were pelleted, and 1 ml of PBS was added. Then, 50 μl of acridine orange (100 μg/ml) was added and mixed in the dark for 15 min. [28] Autophagy was analyzed by flow cytometry (Cytomics FC500 Flow Cytometer, Beckman Coulter).

### Western blot analyses

Cellular lysates were prepared by suspending 1 × 10^6^ cells in 100 μl of RIPA lysis buffer (1 × PBS, 1% NP-40, 0.5% sodium deoxycholate, and 0.1% SDS) that was supplemented with freshly added 10 mM β-glycerophosphate, 1 mM sodium orthovanadate, 10 mM NaF, and 1 mM phenylmethylsulfonyl fluoride. The cells were extracted on ice for 30 min. The proteins were electrotransferred to nitrocellulose membranes (Millipore Corp., Bedford, MA), and detection of the specific proteins was performed using chemiluminescence. All of the antibodies were purchased from Cell Signaling Technology Corporation, Boston, USA.

### Co-IP analysis

The cells were lysed using IP lysis buffer (150 mM NaCl, 25 mM Tris-HCl at pH 7.4, 1 mM EDTA, and 1% NP-40). One mg of protein, 5 μg of antibody (Cell Signaling Technology, USA) and 30 μl of protein A/G agarose beads (Santa Cruz, USA) were added to a 1.5-ml Eppendorf tube, and the tubes were incubated overnight on a rotator at 4°C. The beads were washed and centrifuged, and the supernatant was discarded. Next, 20 μl of 5x loading buffer was added, and the beads were heated for five min at 100°C. Immunoreactive proteins were detected using Western blotting.

### Animal experiment

Female nude mice (4-5 weeks old and weighing 17-23 g) were housed and cared at the animal facility of the Laboratory Animals Centre of Sun Yat-sen University under temperature 22°C and 12 hr light/dark cycle with free access to food and water. All animal maintenance and procedures were carried out in strict accordance with the recommendations established by the Animal Care and Ethics Committee of Sun Yat-sen University as well as the guidelines by the U.S. National Institutes of Health Guide for the Care and Use of Laboratory Animals. The protocol was approved by the Animal Care and Ethics Committee of Sun Yat-sen University. In animal study, all efforts were made to minimize suffering ofmice. All mice were humanely sacrificed by CO_2_ inhalation before death. U937 cells (5×10^6^) were injected subcutaneously into the nude mice. The mice were randomly assigned into two groups: each mouse in the treatment group was injected with 10 mg per kg DAP each day; and an equal volume of PBS solution was injected into the control group (intraperitoneal injection). The length (L) and width (W) of each tumor was measured with calipers, and the volume (V) was calculated as follows: V = (L × W^2^) × 0.5. The volumns of tumors and the weights of mice were measured and calculated for 12 days after treatment. The tumor xenograft weights were measured and deducted from the body weights of mice.

### Statistic analysis

Statistical significance was evaluated using SPSS11.0 software, and *P*<0.05 was considered to be statistically significant. * represents *P*< 0.05, ** represents *P*<0.001, and *** represents *P*< 0.0001.

## References

[1] Smith M, Barnett M, Bassan R, Gatta G, Tondini C, Kern W (2004). Adult acute myeloid leukaemia. Crit Rev Oncol Hematol.

[2] Kindler T, Lipka DB, Fischer T (2010). FLT3 as a therapeutic target in AML: still challenging after all these years. Blood.

[3] Warburg O (1956). On the origin of cancer cells. Science.

[4] Hitosugi T, Kang S, Vander Heiden MG, Chung TW, Elf S, Lythgoe K, Dong S, Lonial S, Wang X, Chen GZ, Xie J, Gu TL, Polakiewicz RD, Roesel JL, Boggon TJ, Khuri FR, Gilliland DG, Cantley LC, Kaufman J, Chen J (2009). Tyrosine phosphorylation inhibits PKM2 to promote the Warburg effect and tumor growth. Sci Signal.

[5] Christofk HR, Vander Heiden MG, Harris MH, Ramanathan A, Gerszten RE, Wei R, Fleming MD, Schreiber SL, Cantley LC (2008). The M2 splice isoform of pyruvate kinase is important for cancer metabolism and tumour growth. Nature.

[6] Langhammer S, Najjar M, Hess-Stumpp H, Thierauch KH (2011). LDH-A influences hypoxia-inducible factor 1α (HIF1-α) and is critical for growth of HT29 colon carcinoma cells in vivo. Target Oncol.

[7] Abu Dawud R, Schreiber K, Schomburg D, Adjaye J (2012). Human embryonic stem cells and embryonal carcinoma cells have overlapping and distinct metabolic signatures. PLoS One.

[8] Silvestri A, Palumbo F, Rasi I, Posca D, Paylidou T, Paoluzi S, Castagnoli L, Cesareni G (2015). Metformin induces apoptosis and downregulates pyruvate kinase M2 in breast cancer cells only when grown in nutrient-poor conditions. PloS One.

[9] Sutendra G, Michelakis ED (2013). Pyruvate dehydrogenase kinase as a novel therapeutic target in oncology. Front Oncol.

[10] Suh DH, Kim MK, Kim HS, Chung HH, Song YS (2012). Unfolded protein response to autophagy as a promising druggable target for anticancer therapy. Ann N Y Acad Sci.

[11] Cao W, Yacoub S, Shiverick KT, Namiki K, Sakai Y, Porvasnik S, Urbanek C, Rosser CJ (2008). Dichloroacetate (DCA) sensitizes both wild-type and over expressing Bcl-2 prostate cancer cells in vitro to radiation. Prostate.

[12] Hur H, Xuan Y, Kim YB, Lee G, Shim W, Yun J, Ham IH, Han SU (2013). Expression of pyruvate dehydrogenase kinase-1 in gastric cancer as a potential therapeutic target. Int J Oncol.

[13] Michelakis ED, Webster L, Mackey JR (2008). Dichloroacetate (DCA) as a potential metabolic-targeting therapy for cancer. Br J Cancer.

[14] Martelli AM, Tazzari PL, Evangelisti C, Chiarini F, Blalock WL, Billi AM, Manzoli L, McCubrey JA, Cocco L (2007). Targeting the phosphatidylinositol 3-kinase/Akt/mammalian target of rapamycin module for acute myelogenous leukemia therapy: from bench to bedside. Curr Med Chem.

[15] Stacpoole PW, Henderson GN, Yan Z, James MO (1998). Clinical pharmacology and toxicology of dichloroacetate. Environmental health perspectives.

[16] Kaufmann P, Engelstad K, Wei Y, Jhung S, Sano MC, Shungu DC, Millar WS, Hong X, Gooch CL, Mao X, Pascual JM, Hirano M, Stacpoole PW, DiMauro S, De Vivo DC (2006). Dichloroacetate causes toxic neuropathy in MELAS: a randomized, controlled clinical trial. Neurology.

[17] Onodera J, Ohsumi Y (2005). Autophagy is required for maintenance of amino acid levels and protein synthesis under nitrogen starvation. J Biol Chem.

[18] Carew JS, Nawrocki ST, Kahue CN, Zhang H, Yang C, Chung L, Houghton JA, Huang P, Giles FJ, Cleveland JL (2007). Targeting autophagy augments the anticancer activity of the histone deacetylase inhibitor SAHA to overcome Bcr-Abl-mediated drug resistance. Blood.

[19] Chen S, Rehman SK, Zhang W, Wen A, Yao L, Zhang J (2010). Autophagy is a therapeutic target in anticancer drug resistance. Biochim Biophys Acta.

[20] Gallay N, Dos Santos C, Cuzin L, Bousquet M, Simmonet Gouy V, Chaussade C, Attal M, Payrastre B, Demur C, Récher C (2009). The level of AKT phosphorylation on threonine 308 but not on serine 473 is associated with high-risk cytogenetics and predicts poor overall survival in acute myeloid leukaemia. Leukemia.

[21] Muranyi AL, Dedhar S, Hogge DE (2009). Combined inhibition of integrin linked kinase and FMS-like tyrosine kinase 3 is cytotoxic to acute myeloid leukemia progenitor cells. Exp Hematol.

[22] Tazzari PL, Cappellini A, Ricci F, Evangelisti C, Papa V, Grafone T, Martinelli G, Conte R, Cocco L, McCubrey JA, Martelli AM (2007). Multidrug resistance-associated protein 1 expression is under the control of the phosphoinositide 3 kinase/Akt signal transduction network in human acute myelogenous leukemia blasts. Leukemia.

[23] Schaich M, Soucek S, Thiede C, Ehninger G, Illmer T (2005). MDR1 and MRP1 gene expression are independent predictors for treatment outcome in adult acute myeloid leukaemia. Br J Haematol.

[24] Park S, Chapuis N, Bardet V, Tamburini J, Gallay N, Willems L, Knight ZA, Shokat KM, Azar N, Viguié F, Ifrah N, Dreyfus F, Mayeux P, Lacombe C, Bouscary D (2008). PI-103, a dual inhibitor of Class IA phosphatidylinositide3-kinase and mTOR, has antileukemic activity in AML. Leukemia.

[25] Gregersen LH, Jacobsen A, Frankel LB, Wen J, Krogh A, Lund AH (2012). MicroRNA-143 down-regulates Hexokinase 2 in colon cancer cells. BMC Cancer.

[26] Elstrom RL, Bauer DE, Buzzai M (2004). Akt stimulates aerobic glycolysis in cancer cells. Cancer Res.

[27] Juntilla MM, Koretzky GA (2008). Critical roles of the PI3K/Akt signaling pathway in T cell Development. Immunol Lett.

[28] Klionsky DJ, Abdalla FC, Abeliovich H, Abraham RT, Acevedo-Arozena A, Adeli K, Agholme L, Agnello M, Agostinis P, Aguirre-Ghiso JA, Ahn HJ, Ait-Mohamed O, Ait-Si-Ali S (2012). Guidelines for the use and interpretation of assays for monitoring autophagy. Autophagy.

[29] White E (2012). Deconvoluting the context-dependent role for autophagy in cancer. Nat Rev Cancer.

[30] Lozy F, Karantza V (2012). Autophagy and cancer cell metabolism. Semin Cell Dev Biol.

[31] Mizushima N (2010). The role of the Atg1/ULK1 complex in autophagy regulation. Curr. Opin. Cell Biol..

[32] Wu W, Tian W, Hu Z, Chen G, Huang L, Li W, Zhang X, Xue P, Zhou C, Liu L, Zhu Y, Zhang X, Li L, Zhang L, Sui S, Zhao B, Feng D (2014). ULK1 translocates to mitochondria and phosphorylates FUNDC1 to regulate mitophagy. EMBO Rep.

[33] Rabinowitz JD, White E (2010). Autophagy and metabolism. Science.

[34] Marquez RT, Xu L (2012). Bcl-2: Beclin 1 complex: multiple, mechanisms regulating autophagy/apoptosis toggle switch. Am J Cancer Res.

[35] Raimondi C, Falasca M (2011). Targeting PDK1 in cancer. Curr Med Chem.

